# High expression of miR-7974 predicts poor prognosis and is associated with autophagy in estrogen receptor-positive breast cancer

**DOI:** 10.1371/journal.pone.0322179

**Published:** 2025-04-29

**Authors:** Stralina Eneh, Jaana M. Hartikainen, Sami Heikkinen, Reijo Sironen, Maria Tengström, Veli-Matti Kosma, Saket Ahuja, Arto Mannermaa

**Affiliations:** 1 Institute of Clinical Medicine, Clinical Pathology and Forensic Medicine, School of Medicine, University of Eastern Finland, Kuopio, Finland; 2 Multidisciplinary Cancer Research Community (Cancer RC), University of Eastern Finland, Kuopio, Finland; 3 Genome Center of Eastern Finland, Institute of Clinical Medicine, School of Medicine, University of Eastern Finland, Kuopio, Finland; 4 Institute of Biomedicine, School of Medicine, University of Eastern Finland, Kuopio, Finland; 5 Department of Clinical Pathology, Kuopio University Hospital, Kuopio, Finland; 6 Cancer Center, Department of Oncology, Kuopio University Hospital, Kuopio, Finland; 7 Biobank of Eastern Finland, Kuopio University Hospital, Kuopio, Finland.; Shanghai Jiaotong University: Shanghai Jiao Tong University, CHINA

## Abstract

Estrogen receptor-positive (ER+) breast cancers (BC) cause death despite well-established treatments. MicroRNAs (miRNAs) have potential as biomarkers specific to cancer subtypes and tissues, therefore miRNA-based biomarkers could help improve patient survival. In this study, we investigated a relatively unknown miRNA, miR-7974. We utilized small RNA data from 204 breast tissue samples to study miR-7974 association with clinicopathological features and outcomes for BC patients. Additionally, *in vitro* and *in ovo* methods were used to identify miR-7974 role at molecular and cellular level in MCF-7 cells. Findings were validated using MDA-MB-453 cells. MiR-7974 was upregulated in many clinicopathological features of BC (*P*<0.05). Furthermore, the highest expression of miR-7974 was associated with poor relapse-free survival in ER+ BC patients [hazard ratio (HR)=8.70; 95% confidence interval (CI)=3.28–23.06; *P*=1.37x10^-05^] and poor BC-specific survival in patients receiving only surgical treatment (HR=8.36; 95% CI=1.01–69.06; *P*=0.049). Our studies revealed that miR-7974 targets autophagy gene, *MAP1LC3B*, identified as direct miR-7974 target (*P*<0.05) in MCF-7 cells. *In vitro* analyses indicated overexpressing miR-7974 had anti-proliferative effect in MCF7 and MDA-MB-453 cells. Overall, our results demonstrate potential prognostic role of miR-7974 in ER+ BC.

## Introduction

Breast cancer (BC) is the most common cancer in women worldwide. BC is divided into molecular subtypes depending on the presence and/or absence of estrogen receptor (ER), progesterone receptor (PR), and human epidermal growth factor receptor 2 (HER2). The hormone receptors are used as biomarkers for estimating prognosis. Thus, the worst prognosis is for a patient with triple-negative (TN; negative for ER, PR, and HER2) BC [1,2]. Although there are well-established treatments for ER-positive (ER+) cases, the disease may still progress and cause death [3]. Therefore, a specific and sensitive biomarker for early detection of BC would improve patient survival. Recently, microRNAs (miRNAs) were suggested as potential biomarkers in BC [4–6].

MiRNAs regulate protein coding genes through post-transcriptional mechanisms, resulting in mRNA degradation and the repression of translation in physiological and pathological processes [7]. In BC, miRNAs are involved in the initiation and progression of BC subtypes [6–9]. MiRNAs control cellular processes, such as cell proliferation, apoptosis, and autophagy, via their target genes [7,10].

Macroautophagy (hereafter, autophagy) is a catabolic recycling process in which cellular components, including proteins and damaged organelles, are transported to the lysosomes for degradation and recycled to be used again for cellular renovation and homeostasis [11–13]. The process of mammalian autophagy has five molecular steps: autophagy induction, vesicle nucleation, vesicle elongation, maturation, and autophagosome fusion with a lysosome [11]. These steps are regulated by several autophagy-related genes, such as the unc51-like autophagy activating kinase (*ULK*) complex, the Beclin 1/class III phosphatidylinositol 3-kinase catalytic subunit type 3 (*PI3KC3*) complex, sequestosome 1 (*SQSTM1*; hereafter, *p62*), microtubule-associated protein 1 light chain 3 beta (*MAP1LC3B*; hereafter, *LC3B*), mammalian target of rapamycin (*mTOR*), and AuTophaGy (*ATG*) [11].

In cancer cells, autophagy has a dual role. It can act as a tumor suppressor by inhibiting cancer cell survival and inducing cell death, but it can also promote tumor development by supporting cancer cell proliferation and tumor growth, especially in advanced stages [14,15]. For instance, knocking down autophagy-related genes like *Beclin-1* and *LC3B* inhibits proliferation and invasion in breast cancer cells [16]. Conversely, mutations that activate *RAS* increase autophagy, leading to enhanced tumor growth, survival, and oncogenesis [14]. This dual role of autophagy makes its influence on cancer treatment complex, as it can both promote and suppress tumors, affecting the effectiveness of therapies in various ways [14]. Additionally, several miRNAs have been shown to be involved in the regulation of autophagy in various diseases, including cancer [11,13]. Although there is a growing interest in autophagy-related miRNAs [11,13], little is known about how miRNAs, especially miR-7974, are associated with autophagy-related genes in cancers.

MiR-7974 was first reported in 2013 in human granulosa cells, where it was predicted to regulate genes involved in morphogenesis and transcription activation using a web-based target prediction algorithm [17]. In addition, using a computational approach, miR-7974 was suggested to be a therapeutic marker in the Middle East Respiratory Syndrome, which is caused by a type of human coronavirus [18]. Furthermore, the expression level of miR-7974 varies in lung cancer subtypes [19] and in invasive oral squamous cell carcinoma [20]. Therefore, miR-7974 seems to play a role in different diseases. Interestingly, *in silico* analysis predicted that miR-7974 is associated with autophagy-related genes. However, since 2013, not much has been published about the functional roles of miR-7974.

In this study, we aimed to characterize the prognostic role of miR-7974 in the long-term follow-up of invasive BC, especially ER+ BC, and its relation to autophagy. We hypothesized that different expression levels of miR-7974 affect the survival of invasive BC patients and the miR-7974 expression pattern observed in clinical data can be seen by studying BC cell lines. In addition, *in vitro* and *in ovo* methods were used to characterize autophagy-related genes in response to miR-7974 upregulation and the functional roles of miR-7974 in MCF-7 cells.

## Materials and methods

### Patient material collection and small RNA-sequencing

The acquisition of patient tissue samples, RNA extraction, sequencing library preparation, RNA and library quality control, and small RNA-sequencing were described previously [21]. Briefly, we used sample material from the Kuopio Breast Cancer Project (KBCP), which was collected from the province of Northern Savo in Eastern Finland and diagnosed at Kuopio University Hospital between April 1990 and December 1995 [22,23]. The KBCP was approved by the Joint Ethics Committee of the University of Eastern Finland and Kuopio University Hospital (reference number 1040/2019). Each patient provided informed written consent for participation in the study. The characteristics of the cases with invasive BC are summarized in S1 Table. Patients involved in this study have not received any neoadjuvant treatments. For this study, we utilized the sequencing data from 182 patients with invasive BC tumors and from 22 patients with benign breast disease.

### Bioinformatic data analysis

Small RNA-Seq pre-processing consisted of read quality assessment using FastQC (v0.10.1; https://www.bioinformatics.babraham.ac.uk/projects/fastqc/), adapter trimming using TRIMMOMATIC (v0.33 with essential parameters: ILLUMINACLIP:TruSeq3-SE.v2.long.fa:0:30:10 LEADING:3 TRAILING:3 MINLEN:20) [24], and removal of reads aligning to ribosomal RNA (GenBank access numbers X12811.1 and U13369.1), the mitochondrial genome (GenBank access number NC_012920.1), phiX174 sensu lato genome (GenBank access number NC_001422.1), and poly-A and poly-C sequences using Bowtie2 (version 2.2.3 [25], with essential parameters: --end-to-end, --sensitive). Pre-processed reads were aligned to the human miRNA transcriptome (miRBase v21) [26] using Tophat (v2.0.13, with essential parameters: -N 1 --read-gap-length 1 --segment-length 40 --segment-mismatches 1 --library-type fr-unstranded --no-coverage-search --b2-sensitive [24] followed by data conversion for visualization (samtools, IGVtools). Gene-wise read counts for miRNAs in miRBase v21 [26] were collected using R function GenomicAlignments::summarizeOverlaps (v1.6.3) [27] in “Union” mode. For uses other than differential gene expression (DEG) analysis, read counts were normalized using varianceStabilizingTransformation in “blind” mode from the R/Bioconductor package DESeq2 (v1.26.0) [28]. Quality control and exploration were performed to find/exclude technical bias (multidimensional scaling, principal component analysis, and unsupervised hierarchical clustering) in R/Bioconductor [29]; no bias assignable to library preparation batch or sequencing run was found.

### Statistical analyses and survival analyses

Significantly differentially expressed (DE) RNAs were identified using the Wald statistical test in R package DESeq2 (v1.26.0), false discovery rate (FDR) for *P*-value adjustment (*P*_adj_), and the R function DESeq2::lfcShrink for shrinking fold changes of RNAs with low expression. The following BC sub-types and clinical variables were tested in the DEG analyses: invasive local BC vs. benign breast tissue, tumor grade II vs. tumor grade I, tumor grade III vs. tumor grade I, tumor grade III vs. tumor grade II, ER negative (ER-) vs. ER+ BC, PR negative (PR-) vs. PR positive (PR+) BC, and TNBC vs. luminal.

Cases with invasive BC (excluding cases with metastases at diagnosis) were included in the survival analyses for relapse-free survival (RFS) and BC-specific survival (BCSS). RFS was defined as the time from diagnosis to the time of first relapse or the end of follow-up. BCSS was defined as the time from the date of BC diagnosis to the date of death due to BC. For survival analysis, BC cases were divided into quartiles by the normalized read count of the small RNA. The lowest quartile was named Q1. Survival analyses were performed by comparing each other quartile of hsa-miR-7976 against Q1. Multivariate survival analyses providing the hazard ratios (HRs) and confidence intervals (CIs) for death or recurrence were performed using the Cox proportional hazards model in a forward stepwise manner in R function MASS::stepAIC v7.3-51.5 [30].

In the multivariate survival analyses, clinical and treatment parameters were used as covariates. Clinical parameters were tumor grade, tumor histology, tumor size, nodal status, ER status, PR status, HER2 status, and age at diagnosis. Treatment parameters were radiotherapy (RT; yes/no), adjuvant chemotherapy (CT; yes/no), and adjuvant hormone therapy (HT; yes/no). Multivariate survival analyses were performed for all cases with invasive local disease and for specific patient groups as follows: ER+ cases (cases with ER+ BC), clinical and treatment parameters were included in the analysis as covariates; ER+ cases, treatment data not included (cases with ER+ BC), only clinical parameters were included in the analysis as covariates; Surgery only cases (BC cases with invasive local BC that did not receive adjuvant RT, CT, or HT), clinical parameters were included in the analysis as covariates. Survival rate plots were generated using basic R plotting functions (v3.6.2). A *P*_adj_-value <0.05 was considered statistically significant in the DEG and multivariate survival analyses.

### *In silico* analysis

The miRNA target prediction algorithm tool TargetScan 7.2 (www.targetscan.org) [31] was used to identify predicted targets of miR-7974. The default settings were used in TargetScan.

### Cell culture

We used authenticated (Multiplexion, Germany) human BC cell line Michigan Cancer Foundation-7 (MCF-7) and MDA-MB-453. For validation purposes, we used the TNBC cell line, MDA-MB-453. The Research Resource Identifier (RRID) as available in the ExPASy Cellosaurus database (https://www.cellosaurus.org/) is RRID:CVCL_0031 for MCF-7 and RRID:CVCL_0418 for MDA-MB-453. In our cell culture laboratory, we frequently confirm that cell lines are free of mycoplasma contamination. MCF-7 cells were cultured in the recommended cell culture medium, Minimum Essential Medium (Gibco) containing 10% fetal bovine serum (Gibco), 1x non-essential amino acid (Gibco), 2 nM L-glutamine (Gibco), 100 U/ml penicillin (Gibco), and 100 mg/ml streptomycin (Gibco). MDA-MB-453 cells were cultered Minimum Essential Medium (Gibco) containing 10% fetal bovine serum (Gibco) and 1% penicillin (Gibco)/streptomycin (Gibco). Cells were kept in an incubator with a humidified atmosphere of 5% CO_2_ at 37^o^C. Antibiotic-free cell culture medium was used for transfections.

### Cell transfection

Human hsa-miR-7974 mirVana™ miRNA mimic (Ambion, Life Technologies) was used to mimic the overexpression of miR-7974. Negative control cells were transfected with Cy3™ Dye-Labeled Anti-miR™ Negative Control #1 and mirVana™ miRNA Mimic Negative Control #1 (Ambion, Life Technologies). For the miRNA pull-down assay, cells were transfected using biotin-labeled hsa-miR-7974 mirVana™ Custom miRNA mimic (Ambion, Life Technologies) and biotin-labeled Neg 1 mirVana™ Custom miRNA mimic (Ambion, Life Technologies).

We seeded MCF-7 and MDA-MB-453 cells into 12-well plates for transfection. MCF-7 cells were seeded into 10-cm diameter and 6-cm diameter Petri dishes to be used for chick chorioallantoic membrane (CAM) assay and miRNA pull-down assay, respectively. Transfections were performed at a concentration of 10 nM miRNA mimic or negative control mimic according to the manufacturer’s instructions using Lipofectamine RNAiMAX Transfection Reagent (Invitrogen).

### Western blot analysis

Seventy-two hours after transfection, we washed the cells twice with 1x PBS before lysing with T-PER Tissue Protein Extraction Reagent (Thermo Scientific) containing 1x Halt Protease Inhibitor Cocktail (Thermo Scientific) to collect the total protein. The lysates were quickly frozen using liquid nitrogen. After thawing them at room temperature and incubating on ice for 30 min, the supernatant fraction containing the proteins was collected by centrifuging for 10 min at 13 000 RPM at 4^o^C. We determined the protein concentration using the Bradford protein assay.

We separated equal amounts of proteins by 10% SDS-PAGE and then transferred the separated proteins to a polyvinylidene difluoride (PVDF) membrane. The PVDF membranes were blocked with 5% non-fat milk in Tris-buffer saline with Tween solution (TBST) for 1 h at room temperature, followed by overnight incubation at 4^o^C with primary antibodies. We used primary antibodies against LC3B [anti-LC3B rabbit polyclonal antibody (ab48394, 1:5000, Abcam)], p62 [anti-p62/SQSTM1 rabbit polyclonal antibody (P0067, 1:5000, Sigma)], and glyceraldehyde-3-phosphate dehydrogenase (GAPDH) [anti-GAPDH mouse monoclonal antibody (ab8245, 1:20 000, Abcam)]. GAPDH was used as a loading control. After overnight incubation, membranes were washed with 1x TBST and incubated with secondary antibodies [donkey anti-Rabbit immunoglobulin G (IgG) (NA934VS, 1:5000, Amersham) for LC3B and p62, and sheep anti-mouse IgG (NA931VS, 1:20 000, Amersham) for GAPDH] for 1.5 h at room temperature. The protein bands were detected using Amersham ECL Prime Western Blotting Detection Reagent (GE Healthcare) and a phosphoimager (FLA3000, Fuji) according to the manufacturer´s protocol. Image processing was done using ImageJ 1.52a software (National Institutes of Health).

### Biotinylated miRNA pull-down

MCF-7 cells (700 000 cells/ 6-cm diameter Petri dish) were transfected with biotin-labeled hsa-miR-7974 or biotin-labeled negative control mimic as described above. Seventy-two hours after transfection, the cells were washed twice with PBS, the cell pellets resuspended in lysis buffer [20 nM Tris (pH 7.5), 100 mM KCL, 5 mM MgCl_2_, 0.3% IGEPAL CA-630 (Sigma-Aldrich), including Halt™ Protease Inhibitor Cocktail (Thermo Scientific) and RNase inhibitor (Applied Biosystems)], and incubated on ice for 20 min. We isolated the cytoplasmic lysate by centrifugation at 18 000 RPM at 4^o^C for 10 min. Approximately 10% of the total volume of cytoplasmic lysate was taken as input RNA samples and the rest of the lysate was used as pull-down RNA.

M-280 Streptavidin beads (Dynabeads, Invitrogen) were activated according to the manufacturer´s instructions. We blocked the beads with lysis buffer containing yeast tRNA (Sigma-Aldrich) for 2 h at room temperature and washed once with 1 ml of lysis buffer. After the washing steps, we added cytoplasmic lysate to the beads and incubated the beads overnight on a rotating shaker at 4^o^C. The next day, we washed the beads three times with 1 ml lysis buffer, transferred the beads to a fresh Eppendorf tube, washed the beads twice, and then transferred the beads to a new Eppendorf tube to minimize any nonspecific pull-down contaminating our final sample.

### RNA isolation

Seventy-two hours after transfection, the total RNA, including miRNA needed for the quantitative real time-PCR (qPCR) analysis, was extracted using the miRNeasy Mini Kit (Qiagen) according to the manufacturer´s instructions. RNA bound to the streptavidin beads (pull-down RNA) and the input RNA were also isolated using the same kit before the qPCR analysis. We measured RNA quality using a UV-Vis Nanodrop 1000 spectrophotometer (Thermo Scientific).

### qPCR

The gene expression levels were quantified using qPCR. For hsa-miR-7974 and hsa-miR-25-3p, as well as peptidylprolyl Isomerase A (*PPIA*), *LC3B,* and *p62*, total RNA was reverse transcribed into cDNA using the miRCURY LNA RT Kit (Qiagen) and High Capacity DNA Reverse Transcription Kit (Applied Biosystems, Thermo Fisher Scientific), respectively, according to the manufacturers´ instructions. qPCR was performed for hsa-miR-7974 and hsa-miR-25-3p using miRCURY LNA PCR Assays (ID: YP02102804 for hsa-miR-7974 and ID: YP00204361 for hsa-miR-25-3p; Qiagen) and miRCURY LNA SYBR Green PCR Kit (Qiagen). Hsa-miR-25-3p was used as the reference. For *PPIA*, *LC3B,* and *p62*, the TaqMan Gene Expression Assay (ID: Hs04194521_s1 for *PPIA*, ID: Hs00797944_s1 for *LC3B* and ID: Hs01061917_g1 for *p62*) and Brilliant III Ultra-Fast QPCR Master Mix (Agilent Technologies) were used. *PPIA* was used as the reference gene. All real-time reactions were run using the LightCycler® 96 Instrument (Roche Life Science). The relative gene expression levels were calculated using the 2^-(∆∆Cq)^ method.

### Cell proliferation analysis

MCF-7 and MDA-MB-453 cells transfected with hsa-miR-7974 and negative mimic were seeded onto 12-well plates (50 000 cells/well) for real-time cell proliferation analysis. The cell confluence was monitored every 3 h for a total of 72 h after transfection using the Incucyte® S3 Live-Cell Analysis System (Essen BioScience).

### CAM assay

Fertilized white Leghorn chicken eggs were used in the CAM assay as described previously [32]. Eggs were incubated at 37^o^C under constant humidity, starting at embryo development day 0 (EDD0). Separation of the CAM was induced on EDD4 by piercing the eggshell. On EDD8, MCF-7 cells (transfected with hsa-miR-7974 mimic and negative control mimic for 72 h) were collected, suspended in PBS and Corning® Matrigel® Matrix GFR Phenol Red Free (1:1; Thermo Fisher Scientific), and implanted on the CAM (10^6^ cells per egg) inside the area framed by a light plastic ring. On EDD13, the tumors were photographed *in ovo* and excised. Tumor area was measured on photographs from 19 eggs for negative control samples and 21 eggs for mimic samples using ImageJ 1.52a (National Institutes of Health).

Tumors were fixed in 3% paraformaldehyde, embedded in paraffin, and cut into 5-µm sections to assess the tumor histology by hematoxylin-eosin (HE) staining as described previously [32]. Stained sections were scanned by a Nanozoomer XR digital slide scanner (Hamamatsu Photonics K.K., Hamamatsu City, Japan) at 40× (kindly provided by the Biobank of Eastern Finland).

### Statistical analysis

The relative protein and gene expression levels were calculated between two groups (miR-7974 mimic transfected cells vs. negative control cells, miR-7974 mimic transfected cells vs. untreated cells, or untreated cells vs. negative control cells). In the cell proliferation assay, proliferation was compared between negative control cells and miR-7974 mimic transfected cells. In the CAM assay, the relative tumor area was calculated (tumor area/ area framed by plastic ring) to assess the tumor growth between the negative control cells and miR-7974 mimic transfected cells. Statistical analyses for different assays were performed in GraphPad Prism 8.4.1 software using the Student´s t-test and data are presented as mean ± standard deviation. A *P*-value <0.05 was considered statistically significant.

## Results

### MiR-7974 is upregulated in clinicopathological features of BC

In the analyses of DEG in the KBCP samples, miR-7974 expression level was significantly (*P*_*adj*_=8.93x10^-06^) higher in invasive local BC cases (n=182) than in benign cases (n=22; Table 1). When the analysis was restricted to locally invasive BC, we found that the association of the expression level of miR-7974 with clinicopathological features was statistically significant (*P*_*adj*_<0.05) in poorly differentiated BC (Table 1). For example, miR-7974 was upregulated in grade II (n=85) and III (n=66) tumors compared to grade I (n=31) tumors, which remained true when the analyses were adjusted for ER status, meaning that the association was independent of ER status. In addition, miR-7974 was upregulated in ER-, PR- and TNBC cases compared to ER+, PR+ and luminal BC cases, respectively.

**Table 1:** miR-7974 is significantly upregulated in clinicopathological features of BC.

	Tumor characteristics					Adjusted by ER status	
	**Comparison**	**vs.**	**Reference**	**Log**_**2**_ **(FC)** ^a^	***Padj*** ^b^	**Log**_**2**_ **(FC)** ^a^	***Padj*** ^b^
	Invasive local		Benign	1.96	8.93x10^-06^	NA	NA
	(n=182)		(n=22)				
Invasive local BC	Grade II		Grade I	1.09	0.009	0.98	0.012
	(n=85)		(n=31)				
	Grade III		Grade I	2.30	4.57x10^-12^	1.87	4.08x10^-07^
	(n=66)		(n=31)				
	Grade III		Grade II	1.20	2.91x10^-06^	0.89	0.006
	(n=66)		(n=85)				
	ER-		ER+	1.23	1.62x10^-05^	NA	NA
	(n=51)		(n=130)				
	PR-		PR+	0.87	0.004	NA	NA
	(n=75)		(n=106)				
	TNBC		Luminal	1.12	0.001	NA	NA
	(n=33)		(n=130)				

^a^The Log2-transformed fold change in the differential expression

^b^The FDR-adjusted P-value for the fold change. *P*adj<0.05 was considered statistically significant.

Abbreviations: FC, fold change; *P*adj, adjusted P-value; NA, not applicable; ER, estrogen receptor; PR, progesterone receptor; TNBC, triple-negative breast cancer

The number of patients in each group is indicated with n in brackets. ER and PR refers to the receptor status of the tumor.

### miR-7974 is a potential prognostic marker in ER+ BC

**Fig 1:**
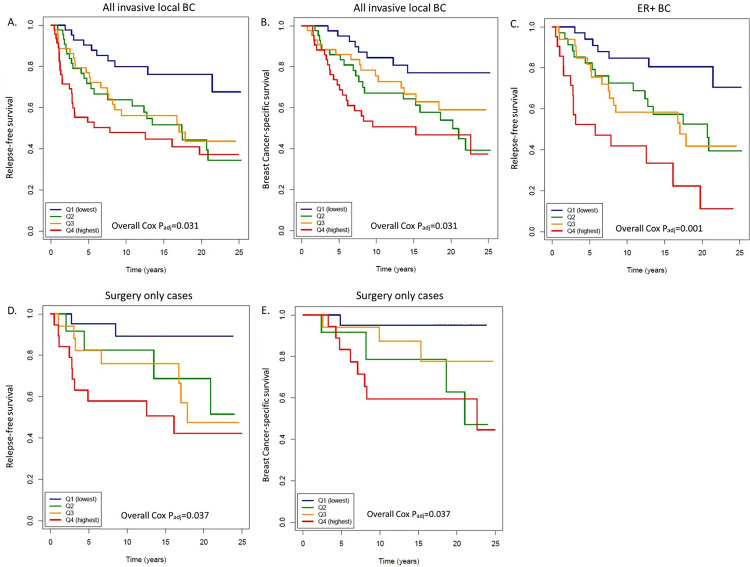
High expression of miR-7974 is associated with poor prognosis in invasive local BC in Cox multivariate analysis. **(A)** The highest expression of miR-7974 (Q4) was significantly associated with poorer RFS [*P*=0.003, HR (CI 95%) = 3.11(1.46-6.64)] compared to the lowest expression (Q1) of miR-7974 in all invasive local cases (n=170). **(B)** The highest expression of miR-7974 (Q4) was significantly associated with poorer BCSS [*P*=0.004, HR (CI 95%) = 3.61(1.52-8.62)] compared to the lowest expression (Q1) of miR-7974 in all invasive local cases (n=170). **(C)** Higher expression of miR-7974 (Q2, Q3, Q4) was significantly associated with poorer RFS [*P*=0.037, HR (CI 95%) = 2.58(1.06-6.29) for Q2, *P*=0.006, HR (CI 95%) = 3.63 (1.44-9.15) for Q3 and *P*= 1.37E-05, HR (CI 95%) = 8.70 (3.28-23.06) for Q4] compared to the lowest expression (Q1) in the invasive local ER+ cases (n=120). **(D)** The highest expression of miR-7974 (Q4) was significantly associated with poorer RFS [*P*=0.001, HR (CI 95%) = 19.05(3.25-111.46)] compared to the lowest expression (Q1) in the cases who received only surgery (n=66). **(E)** The highest expression of miR-7974 (Q4) was significantly associated with poorer BCSS [*P*=0.049, HR (CI 95%) = 8.36(1.01-69.06)] compared to the lowest expression (Q1) in the cases who received only surger y (n=66).

According to the Cox multivariate survival analyses in which clinical and treatment parameters were used as covariates, the highest expression of miR-7974 (Q4) was significantly (*P*_*adj*_<0.05) associated with poorer RFS and BCSS compared to the lowest expression (Q1) in all invasive local BC cases (n=170; Table 2, Figs 1A and B). MiR-7974 was identified as a potential prognostic marker in ER+ BC, as the higher expression quartiles of miR-7974 (Q2, Q3 and Q4) were associated with poorer RFS compared to the lowest expression quartile (Q1) in ER+ BC cases (n=120; Table 2, Fig 1C) with *P*_*adj*_=0.001. When treatment parameters were omitted as covariates from the analyses, the highest expression of miR-7974 (Q4) was also statistically significantly (*P*_*adj*_=0.001) associated with poorer RFS in invasive local ER+ cases (n=120; Table 2). Furthermore, the highest expression of miR-7974 (Q4) was significantly (*P*_*adj*_<0.05) associated with poorer RFS and BCSS in patients who received only surgical treatment (n=66; Table 2, Figs 1D and E).

**Table 2:** High expression of miR-7974 is associated with worse patient outcome in the Cox multivariate survival analysis of invasive local BC.

Case	Survival type	*Padj* ^b^	*P* ^c^			HR (CI 95%)		
**Q2**	**Q3**	**Q4**	**Q2**	**Q3**	**Q4**
**all invasive local cases (n=170)**	RFS	**0.031**	0.053	0.126	**0.003**	2.11(0.99–4.49)	1.85(0.84–4.05)	3.11(1.46–6.64)
	BCSS	**0.031**	0.103	0.236	**0.004**	2.02(0.87–4.71)	1.72(0.70–4.24)	3.61(1.52–8.62)
**ER+ cases**^**a**^ **(n=120)**	RFS	**0.001**	**0.037**	**0.006**	**1.37E-05**	2.58(1.06–6.29)	3.63 (1.44–9.15)	8.70 (3.28–23.06)
**ER+ cases**^**a**^ **(n=120),** treatment parameters were not included as covariates	RFS	**0.001**	0.175	0.072	**0.004**	1.88(0.76–4.67)	2.39(0.92–6.17)	4.76(1.63–13.91)
**Surgery only cases (n=66)**	RFS	**0.037**	0.068	0.096	**0.001**	4.95(0.89–27.55)	4.04(0.78–20.82)	19.05(3.25–111.46)
	BCSS	**0.037**	0.055	0.426	**0.049**	8.61(0.95–77.62)	2.60(0.25–27.28)	8.36(1.01–69.06)

Clinical parameters (age at diagnosis, grade, tumor size, nodal status, ER-, PR-, HER2 status, and histology) and treatment parameters (CT, HT, and/or RT) were included as covariates in the analyses in addition to the miRNA-7974 expression quartiles. Statistically significant *P*-values (*P*_adj_<0.05 and Q vs. Q1 *P*<0.05) are in bold.

^a^ER status was not included as a covariate in the analysis.

^b^The false discovery rate-adjusted overall *P*-value.

^c^In the Cox multivariate analysis, the overall *P*-value for the significance of the difference in patient outcome for the miR-7974 expression quartile compared to Q1.

Abbreviations: BCSS, death due to breast cancer; CI, confidence interval; CT, chemotherapy; ER+, estrogen receptor-positive; HR, hazard ratio; HT, hormone therapy; *P*adj, adjusted P-value; RFS, breast cancer recurrence; RT, radiotherapy.

### *In silico* analysis supports autophagy-related genes as targets of hsa-miR-7974

We used TargetScan [31] to determine what genes are predicted to be targets of miR-7974. A total of 4578 predicted target genes were listed for hsa-miR-7974 in TargetScan (S2 Table). Interestingly, the *in silico* analysis indicated a connection of mir-7974 with autophagy. At least 26 autophagy-related genes [11,13] were identified from the predicted targets (S2 Table). One of the important autophagy marker genes, *MAP1LC3B*, was listed as a target of miR-7974. Other autophagy-related genes, including *MTOR*, *ATG5, ATG7,* and *ATG13* [11,13], were also predicted as targets of miR-7974 by TargetScan.

### Autophagy markers p62 and LC3BI are downregulated in MCF-7 cells overexpressing miR-7974

**Fig 2:**
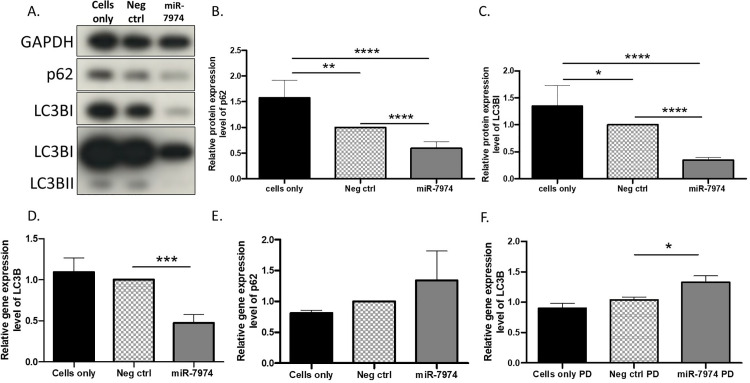
Protein and gene expression levels of p62 and LC3B in miR-7974 mimic-transfected MCF-7 cells. **(A)** The expression levels of p62, LC3BI, and LC3BII were evaluated by Western blot analysis 72 h after miR-7974 transfection of MCF-7 cells. Representative western blots were cropped from respective western blot images for the protein of interest and grouped together. **(B)** The fold change of p62 and **(C)** LC3BI between negative control/cell-only samples and miR-7974-treated samples were statistically significant. GAPDH was used as a reference protein. Seventy-two hours after cell transfection with the miR-7974 mimic, RT-qPCR was performed. **(D)** The gene expression level of *LC3B* was decreased and **(E)** expression level of *p62* upregulated in miR-7974-treated MCF-7 cells. **(F)** The streptavidin pull-down enriched *LC3B*, a target of miR-7974 in MCF-7 cells. Cells were transfected with the bi-neg-ctrl or bi-miR-7974 mimics. Seventy-two hours after mimic transfection, RT-qPCR was performed. The gene expression level of *LC3B* was increased in the pull-down samples. For the gene expression analysis, *PPIA* was used as a reference gene. Statistical analysis was unpaired two-tailed t-test. *P < 0.05, **P < 0.01, ***P < 0.001, ****P < 0.0001, N = 6 (Western blot analysis) and N=3 (gene expression analysis). Full length western blots for the Fig 2A have been provided under supplementary figures, S1 raw image. and S2 raw image.

To study the effect of miR-7974 on the autophagy pathway, we used Western blot analysis to measure p62 and LC3BI protein expression levels in MCF-7 cells 72 h after miR-7974 mimic transfection. Additionally, since there were significant associations with the expression levels of miR-7974 in ER+ breast cancer (Table 2), we chose MCF-7 cells as our ER+ cell line. The protein expression levels of p62 and LC3BI were decreased in miR-7974 mimic-transfected MCF-7 cells compared to negative control cells and untreated cells (*P*<0.0001; Figs 2A–C).

### miR-7974 overexpression correlates with upregulation of *p62* expression level and downregulation of *LC3B* expression level

Next, we investigated the impact of miR-7974 overexpression on autophagy-related genes *p62* and *LC3B* using qPCR. In qPCR analysis, the expression level of *LC3B* was significantly decreased in miR-7974 mimic transfected cells compared to the negative control cells and untreated cells (*P*<0.001; Fig 2D). The expression level of *p62* did not show any significant differences between the groups (Fig 2E), indicating that *p62* is not downregulated by miR-7974.

### *LC3B* is a direct target of miR-7974

Based on our results, overexpression of miR-7974 decreased the expression level of *LC3B* (Fig 2D). To investigate whether *LC3B* is a direct target of miR-7974, we performed streptavidin pull-down for Bi-miR-7974 72 h after the transfection of biotinylated miR-7974 mimic into MCF-7 cells. The expression level of *LC3B* increased in the miR-7974 pull-down samples compared to the negative control samples (Fig 2F). The enrichment of *LC3B* expression in the pull-down of Bi-miR-7974 validates *LC3B* as a direct target of miR-7974 (Fig 2F).

### miR-7974 has an antiproliferative effect on MCF-7 cells

**Fig 3:**
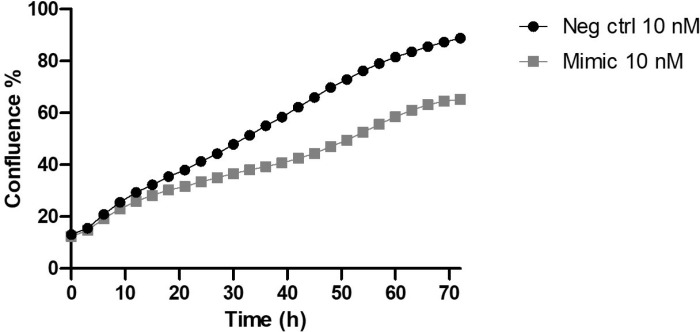
Comparison of proliferation between MCF-7 cells transfected with miR-7974 and control mimics. MCF-7 cells were transfected using miR-7974 mimic (10 nM) and negative control (10 nM). The difference in proliferation between the two groups was statistically significant (*P*=0.020). N=5.

To evaluate the effect of miR-7974 overexpression on the proliferation of MCF-7 cells, we transfected miR-7974 mimic into MCF-7 cells. After 72 hours, we observed significantly (*P*=0.020) slower proliferation in MCF-7 cells overexpressing miR-7974 than in the negative control cells (Fig 3).

### Overexpression of miR-7974 has a suppressive effect on the growth of CAM tumors

**Fig 4:**
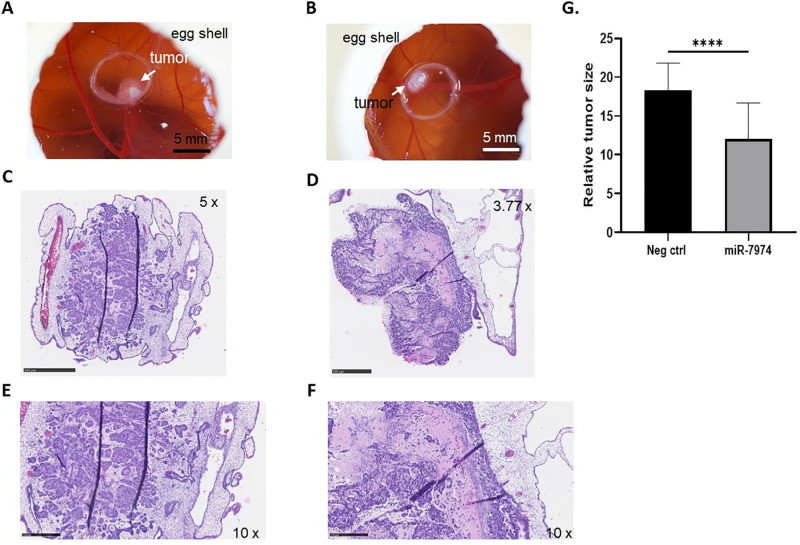
Examples of typical CAM tumors (EDD13). **(A)** Grown from MCF-7 negative control and **(B)** MCF-7 mimic transfected cells. **(C)** HE staining of the whole sections from tumors grown from MCF-7 negative control (5x magnification, scale bar = 500 µm) and **(D)** MCF-7 mimic (3.77x magnification, scale bar = 500 µm) cells. **(E)** Higher magnification (10x, scale bar = 250 µm) and insert (40x, scale bar = 100 µm) of HE staining shows the tumor histology for the tumors grown from MCF-7 negative control and **(F)** MCF-7 mimic cells. **(G)** A statistically significant difference was detected in the size of the negative control and miR-7974 mimic tumors. ****P < 0.0001. N = 19 for control, N = 21 for miR-7974.

To determine if overexpression of miR-7974 has a similar effect on 3D models as in MCF-7 cells, miR-7974 mimic-transfected MCF-7 cells and negative control MCF-7 cells were transplanted onto the CAM membrane of fertilized eggs, resulting in tumor formation (Figs 4A and B). There were no differences in the histopathological features between the samples in HE-stained slides (Figs 4C–F). However, the tumor size (area) was significantly (*P*<0.0001) smaller in the CAM tumors grown from MCF-7 miR-7974 mimic-transfected cells compared to the CAM tumors grown from MCF-7 negative control cells (Fig 4G).

### Validation of miR-7974 mediated targeting of *LC3B* in MDA-MB-453 cells

We identified that *LC3B* is a direct target of miR-7974, and its gene expression and protein levels showed downregulation in MCF-7 cells when transfected with miR-7974 mimic. We aimed to validate these results from MCF-7 cells in a TNBC cell line, MDA-MB-453, since a significant upregulation of miR-7974 was observed in ER-, PR- and TNBC patients (Table 1). To achieve this, we measured total LC3B protein levels and *LC3B* gene expression levels in MDA-MB-453 cells at 72 h after transfection with the miR-7974 mimic. Additionally, we also investigated the effect of miR-7974 overexpression on the proliferation potential of MDA-MB-453 cells by monitoring cell growth over the 72-hour period following transfection.

**Fig 5:**
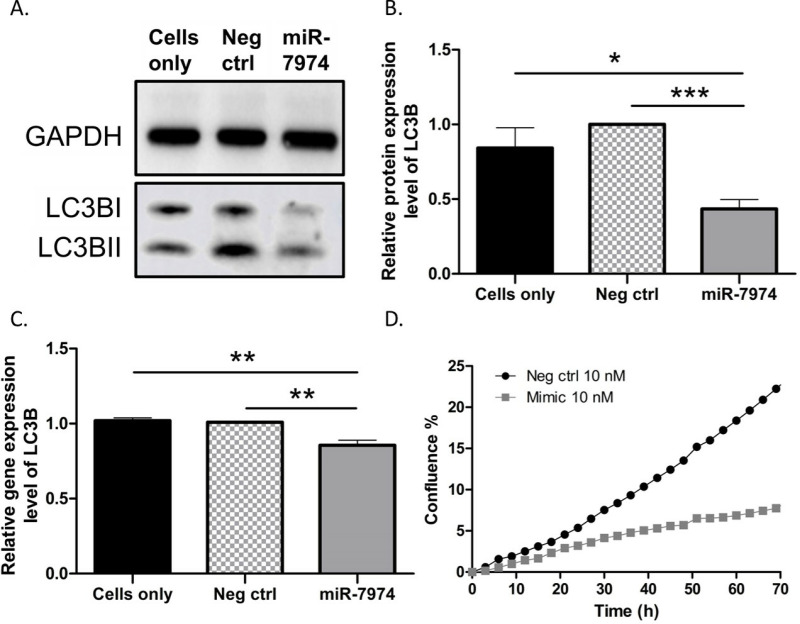
Effect of miR-7974 mimic transfection on proliferation of MDA-MB-453 cells together with changes in protein and gene expression levels of LC3B. **(A)** The protein expression level of LC3BI and LC3BII were evaluated by Western blot analysis 72 h after miR-7974 transfection of MDA-MB-453 cells. Representative western blots were cropped from respective western blot images for the protein of interest and grouped together. GAPDH was used as a reference protein. **(B)** The fold change of total protein levels of LC3B between negative control/cells only samples and miR-7974-treated samples were statistically significant. **(C)** The gene expression levels of *LC3B* were decreased 72h after miR-7974 transfection of MBA-MB-453 cells compared to the negative control/cells-only samples. *PPIA* was used as a reference gene. **(D)** MDA-MB-453 cells were transfected using miR-7974 mimic (10 nM) and negative control (10 nM). The difference in proliferation between the two groups was statistically significant (P=0.0005). Statistical analysis for western blot analysis, gene expression analysis and proliferation analysis were unpaired two-tailed t-test. *P < 0.05, **P < 0.01, ***P < 0.001, ****P < 0.0001, N = 6 (Western blot analysis), N=6 (gene expression analysis) and N=3 (proliferation analysis). Full length western blots for the Fig 5A have been provided under supplementary figure (S3 raw image.).

The protein levels of LC3B were significantly reduced in MDA-MB-453 cells transfected with the miR-7974 mimic compared to both negative control cells and untreated cells (*P*<0.001 and *P*<0.05, respectively; Figs 5A and B), supporting the findings observed in MCF-7 cells. Similarly, qPCR analysis revealed a significant decrease in the expression levels of *LC3B* gene in miR-7974 mimic transfected MDA-MB-453 cells compared to the negative control transfected cells and untreated cells (*P*<0.01; Fig 5C), as observed in MCF-7 cells. Furthermore, like MCF-7 cells, we observed a significantly slower proliferation in MDA-MB-453 cells overexpressing miR-7974 compared to the negative control transfected cells (*P*=0,0005, Fig 5D).

## Discussion

We found that miR-7974 is associated with many clinicopathological features of BC. MiR-7974 was upregulated in higher-grade tumors, and the expression pattern of miR-7974 varied across BC subtypes. In addition, miR-7974 was upregulated in ER-, PR-, and TNBC cases. Higher expression of miR-7974 was associated with poorer RFS of ER+ BC cases, and poor RFS and BCSS was evident in patients who received only surgical treatment. Even though the hormone receptors are currently used to classify the subtypes of BC and to evaluate patient prognosis, miRNAs can play a promising role in classifying BC and estimating survival in BC patients [1,33,34]. Our results based on the clinical data support the conclusion that miR-7974 is a potential marker for the molecular classification of BC into ER+ BC and TNBC, and it may also be a prognostic biomarker in ER+ BC.

Recent studies have highlighted the role of miR-7974 in various diseases. Chen et al. identified miR-7974 as a potential biomarker for lupus nephritis using bioinformatics approaches [35]. Another study by Zheng et al. predicted that *FKBP15* is a target gene of miR-7974 in dental follicle stem cells, specifically in endocytosis [36]. Identifying miR-7974 target genes is key to understanding its physiological role and functions. Although TargetScan predicted several autophagy-related genes, including *MTOR, ATG5, ATG7*, and *ATG13*, as targets of miR-7974, we focused on *LC3B* and *p62*. These genes are widely used as markers to monitor autophagic flux and play key roles in autophagosome formation [37,38]. LC3B is essential for autophagosome formation and maturation, forming LC3B-II, which integrates into the autophagosomal membrane [37,38], whereas p62 acts as a selective receptor, aiding in the degradation of ubiquitinated protein aggregates within autophagosomes [37,38].

Predicted target genes should be confirmed *in vitro* because bioinformatics target prediction tools suffer from high false-positive targets (~30–70%) [39,40]. In this study, we used the biotinylated miRNA pull-down method, which has high sensitivity and low false-positive rate, to find a direct target gene of miRNA. The biotinylated pull-down method has some generic limitations; instead of using the cell’s own miRNA regulation processes, overexpression of miRNA mimics can affect cellular transcription, causing changes in the mRNA levels of certain genes [41]. We used a lower concentration of miRNA mimic and transfection reagent to overcome changes in the expression levels of different mRNAs, which could be due to the overexpression of the chosen miRNA or use of transfection reagents. In this way, we may avoid changes in the levels of mRNA that are not the actual targets of the miRNA in question. In addition, to avoid non-specific binding between exogenous miRNAs and mRNAs [42], we used biotin-labeled negative controls in the pull-down assay. Furthermore, we precisely performed adequate washing and blocking step, as they are important for minimizing the background noise and increasing target specificity and sensitivity of miRNA [42].

We identified *LC3B*, which encodes one of the key proteins in the autophagy pathway [11,13], as a direct target of miR-7974 in MCF-7 cells. Mikhaylova et al. showed that *LC3B* is a direct target of miR-204 in renal clear cell carcinoma [43]. Downregulation of LC3B prevents autophagy and inhibits growth of renal clear cell carcinoma under *in vitro* conditions and *in vivo* in mice [43]. Our functional studies showed that MCF-7 cell proliferation *in vitro* was slower and tumor growth was reduced in cells overexpressing miR-7974 under *in ovo* conditions. We suggest that miR-7974 binds to *LC3B*, thereby decreasing the LC3B protein level, which disturbs the autophagy pathway, possibly inhibiting cell proliferation and tumor growth. Knockdown of *MAP1LC3B* or direct inhibition of autophagy has been shown to result in decreased cancer cell proliferation under *in vitro* settings [44,45]. Detailed description of the effects of this autophagy pathway inhibition would require further studies but a decrease in proliferation rate of cancer cells can have an impact on the effect of cancer treatment strategies.

In the early stages of cancer development, autophagy acts as a cellular quality-control mechanism by maintaining cellular homeostasis, eliminating damaged proteins and organelles that accumulate during stress, thereby inhibiting the growth of tumors [12,46]. We found that CAM tumors grown from miR-7974-transfected MCF-7 cells were smaller than those grown from the negative control samples. The smaller tumor size could be due to the knockdown of *LC3B* caused by miR-7974 overexpression, resulting in decreased proliferation due to inhibition of autophagy. A slower rate of cancer stem cell proliferation helps avoid cell death via drugs targeting rapidly dividing cells [47]. These cancer cells are then more aggressive and more resistant to anti-cancer treatments previously administered to these patients, resulting in a poor long term survival rates in patients with an increased miR-7974 expression levels. Studies have shown that inhibition of autophagy by certain drugs can increase the sensitivity of the cancer cells to anti-cancer drugs, indicating the role of autophagy in cancer drug-resistance mechanisms [48]. In our CAM model, the tumors grew for 5 days after the miR-7974 mimic-treated MCF-7 cells were placed on the CAM. Although the CAM model allows us to study how cancer cells would behave under *in ovo* conditions, the time frame for the study was short compared to the time frame for tumor development in patients. Thus, the CAM model may represent an early stage of tumor but not be a model for well-developed late-stage tumors in patients from whom the clinical data for this study were derived. In addition, upregulation of autophagy proteins, such as p62 and LC3B, is associated with defective autophagy, promoting tumorigenesis [49,50]. In our study, the p62 and LC3B proteins were downregulated in MCF-7 cells overexpressing miR-7974, resulting in decreased autophagic activity, which may have caused the small size of the miR-7974-treated CAM tumors. Autophagy in the late stage of tumorigenesis promotes the growth of cancerous tumors by preventing cancer cell death under stressful conditions [12,49]. In our clinical data, a long follow-up period (nearly 25 years) provided a great opportunity to study the association of high expression of miR-7974 with the survival of BC patients. Furthermore, the autophagy pathway may be one of the mechanisms underlying the effect of miR-7974 overexpression, promoting the tumorigenesis in these patients, subsequently resulting in poor survival.

We identified miR-7974 mediated targeting of *LC3B* in both MDA-MB-453 and MCF-7 cells. The protein levels of LC3B and the *LC3B* gene expression levels were reduced in miR-7974 mimic transfected cells compared to controls in both cell lines. Additionally, we noted a decreased rate of cell proliferation in both MCF-7 and MDA-MB-453 cell lines overexpressing miR-7974 as compared to the negative control. These results confirm that miR-7974 targets and represses *LC3B* gene expression in a cell line-independent manner, indicating that this effect would be consistent across different cell types.

## Conclusions

To summarise, we observed that high expression of miR-7974 in the tumor at diagnosis is associated with ER+ BC and TNBC and the poor survival of patients with invasive BC. The dual role of autophagy can explain the underlying mechanism of poor patient survival and a tumor suppressive effect under *in vitro* and *in ovo* conditions in combination with higher expression levels of mir-7974. We also present *LC3B* as one of the direct targets of miR-7974 thereby indicating the role of miR-7974 in autophagy pathway regulation. Further studies are needed to better understand the role of miR-7974 in autophagy pathway regulation. Our results also suggest miR-7974 as a potential prognostic biomarker for ER+ BC.

## Supporting information

S1 Table Clinical characteristics of patients with invasive breast cancer. (XLSX)

S2 Table Prediction of target genes of hsa-miR-7974 using TargetScan. A total of 4578 predicted target genes were listed. Some of the autophagy-related genes including *MAP1LC3B* were highlighted with yellow color in the table.
(XLSX)

S1 raw image Representative GAPDH, p62 and LC3BI blots shown in Fig 2A. Blots were taken from the third set of samples shown in S1 raw image, indicated at the bottom right of the S1 raw image. This figure contains blots for p62, GAPDH and LC3B proteins developed from three biological replicates. Protein samples used in this western blot were isolated from MCF-7 cells untransfected, transfected with negative control mimic miRNA and miR-7974 mimic transfected. PVDF membrane was cut horizontally based on the size of target protein (kDa) to allow us to measure all proteins from same set of samples at the same time. This image was developed after 10 seconds exposure to the chemiluminescent PVDF membrane.
(PDF)

S2 raw image Representative LC3BI and LC3BII blot shown in Fig 2A. Blot was taken from the third set of samples shown in S2 raw image, indicated by an arrow at the bottom right of the figure. This blot was exposed longer than the blot used in S1 raw image to be able to see LC3BII band because at lower exposures, LC3BII band was not visible. This figure contains blots for p62, GAPDH, LC3BI and LC3BII proteins developed from three biological replicates. Protein samples used in this western blot were isolated from MCF-7 cells untransfected, transfected with negative control mimic miRNA and miR-7974 mimic transfected. PVDF membrane after transfer of proteins was cut horizontally based on the size of protein (kDa) to allow us to measure all proteins from same samples at the same time. For overexposure and visibility of the LC3BII band, this image was developed after 60 seconds exposure to the chemiluminescent PVDF membrane
(PDF)

S3 raw image Representative GAPDH and LC3B blots shown in Fig 5A. Blots were taken from the second set of samples (BR-2) shown in S3 raw image. This figure contains blots for LC3B and GAPDH proteins developed from three biological replicates. Protein samples used in this western blot were isolated from MDA-MB-453 cells untransfected, transfected with negative control mimic miRNA and miR-7974 mimic transfected. PVDF membrane was cut horizontally based on the size of target protein (kDa) to allow us to measure all proteins from same set of samples. Image for LC3B was developed after 30 seconds exposure to the chemiluminescent PVDF membrane and the image for GAPDH was developed after 60 seconds exposure to the chemiluminescent PVDF membrane.
(PDF)
