# Supplementary material for: High expression of miR-7974 predicts poor prognosis and is associated with autophagy in estrogen receptor-positive breast cancer
Source: PLoS One. 2025 Apr 29;20(4):e0322179. doi: 10.1371/journal.pone.0322179 (PMC12040258; doi:10.1371/journal.pone.0322179)
Supplement: S1 raw image — Blots were taken from the third set of samples shown in S1 raw image, indicated at the bottom right of the S1 raw image. This figure contains blots for p62, GAPDH and LC3B proteins developed from three biological replicates. Protein samples used in this western blot were isolated from MCF-7 cells untransfected, transfected with negative control mimic miRNA and miR-7974 mimic transfected. PVDF membrane was cut horizontally based on the size of target protein (kDa) to allow us to measure all proteins from same set of samples at the same time. This image was developed after 10 seconds exposure to the chemiluminescent PVDF membrane. (PDF) [file pone.0322179.s003.pdf]

Supplementary Figures

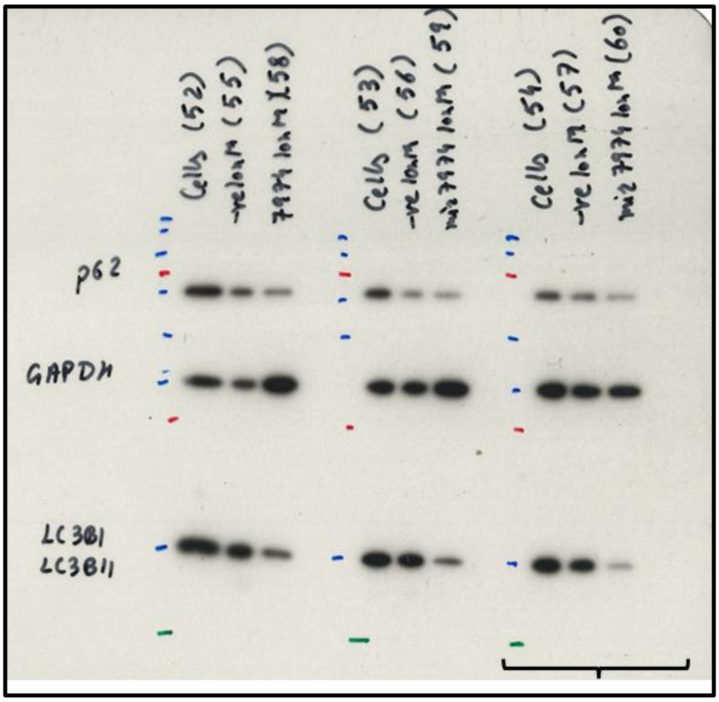

S1 raw image. Representative GAPDH, p62 and LC3BI blots shown in Fig 2A. Blots were taken from the third set of samples shown in S1 raw image, indicated at the bottom right of the S1 raw image. This figure contains blots for p62, GAPDH and LC3B proteins developed from three biological replicates. Protein samples used in this western blot were isolated from MCF-7 cells untransfected, transfected with negative control mimic miRNA and miR-7974 mimic transfected. PVDF membrane was cut horizontally based on the size of target protein (kDa) to allow us to measure all proteins from same set of samples at the same time. This image was developed after 10 seconds exposure to the chemiluminescent PVDF membrane.
