# Supplementary material for: High expression of miR-7974 predicts poor prognosis and is associated with autophagy in estrogen receptor-positive breast cancer
Source: PLoS One. 2025 Apr 29;20(4):e0322179. doi: 10.1371/journal.pone.0322179 (PMC12040258; doi:10.1371/journal.pone.0322179)
Supplement: S2 raw image — Blot was taken from the third set of samples shown in S2 raw image, indicated by an arrow at the bottom right of the figure. This blot was exposed longer than the blot used in S1 raw image to be able to see LC3BII band because at lower exposures, LC3BII band was not visible. This figure contains blots for p62, GAPDH, LC3BI and LC3BII proteins developed from three biological replicates. Protein samples used in this western blot were isolated from MCF-7 cells untransfected, transfected with negative control mimic miRNA and miR-7974 mimic transfected. PVDF membrane after transfer of proteins was cut horizontally based on the size of protein (kDa) to allow us to measure all proteins from same samples at the same time. For overexposure and visibility of the LC3BII band, this image was developed after 60 seconds exposure to the chemiluminescent PVDF membrane (PDF) [file pone.0322179.s004.pdf]

Supplementary Figures

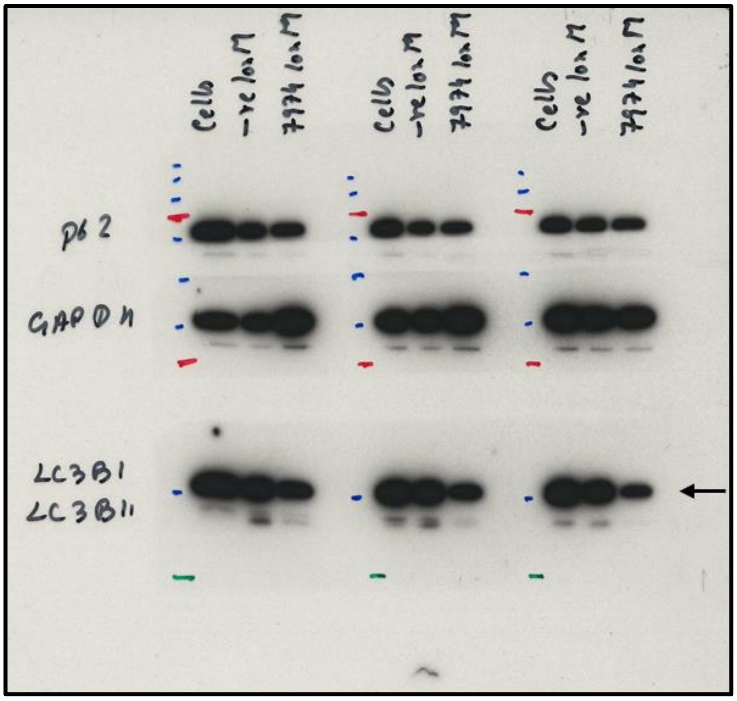

S2 raw image. Representative LC3BI and LC3BII blot shown in Fig 2A. Blot was taken from the third set of samples shown in S2 raw image., indicated by an arrow at the bottom right of the figure. This blot was exposed longer than the blot used in S1 raw image to be able to see LC3BII band because at lower exposures, LC3BII band was not visible. This figure contains blots for p62, GAPDH, LC3BI and LC3BII proteins developed from three biological replicates. Protein samples used in this western blot were isolated from MCF-7 cells untransfected, transfected with negative control mimic miRNA and miR-7974 mimic transfected. PVDF membrane after transfer of proteins was cut horizontally based on the size of protein (kDa) to allow us to measure all proteins from same samples at the same time. For overexposure and visibility of the LC3BII band, this image was developed after 60 seconds exposure to the chemiluminescent PVDF membrane.
