# Supplementary material for: High expression of miR-7974 predicts poor prognosis and is associated with autophagy in estrogen receptor-positive breast cancer
Source: PLoS One. 2025 Apr 29;20(4):e0322179. doi: 10.1371/journal.pone.0322179 (PMC12040258; doi:10.1371/journal.pone.0322179)
Supplement: S3 raw image — Blots were taken from the second set of samples (BR-2) shown in S3 raw image. This figure contains blots for LC3B and GAPDH proteins developed from three biological replicates. Protein samples used in this western blot were isolated from MDA-MB-453 cells untransfected, transfected with negative control mimic miRNA and miR-7974 mimic transfected. PVDF membrane was cut horizontally based on the size of target protein (kDa) to allow us to measure all proteins from same set of samples. Image for LC3B was developed after 30 seconds exposure to the chemiluminescent PVDF membrane and the image for GAPDH was developed after 60 seconds exposure to the chemiluminescent PVDF membrane. (PDF) [file pone.0322179.s005.pdf]

Supplementary Figures

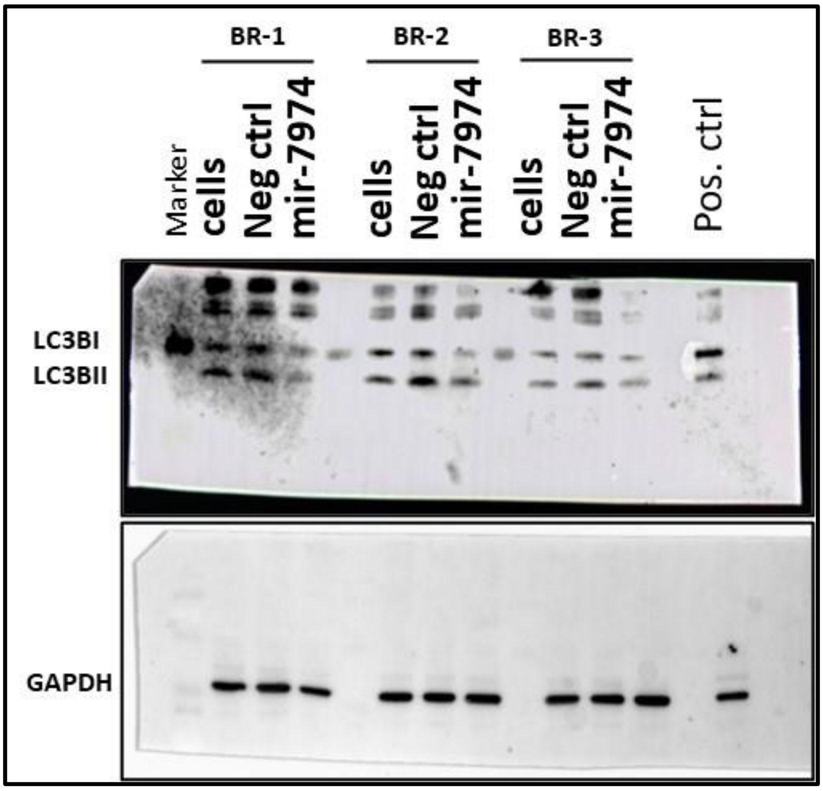

S3 raw image. Representative GAPDH and LC3B blots shown in Fig 5A. Blots were taken from the second set of samples (BR-2) shown in S3 raw image. This figure contains blots for LC3B and GAPDH proteins developed from three biological replicates. Protein samples used in this western blot were isolated from MDA-MB-453 cells untransfected, transfected with negative control mimic miRNA and miR-7974 mimic transfected. PVDF membrane was cut horizontally based on the size of target protein (kDa) to allow us to measure all proteins from same set of samples. Image for LC3B was developed after 30 seconds exposure to the chemiluminescent PVDF membrane and the image for GAPDH was developed after 60 seconds exposure to the chemiluminescent PVDF membrane.
